# Embedding ethics up front in AI and robotics: evidence from future engineers

**DOI:** 10.1007/s43681-026-00991-x

**Published:** 2026-02-01

**Authors:** Anne-Marie Oostveen, Iveta Eimontaite

**Affiliations:** Centre for Robotics and Assembly, Faculty of Engineering and Applied Sciences (FEAS), Cranfield University, Cranfield, UK

**Keywords:** AI ethics, Ethics education, Professional codes of ethics, Ethics up front, Governance of AI, Emerging technologies

## Abstract

As artificial intelligence and robotics increasingly shape societies, ensuring that these technologies align with ethical and societal values is a pressing challenge. This paper presents survey findings from 98 MSc Robotics and Applied AI students at Cranfield University, offering rare empirical evidence of how future AI and robotics professionals perceive their ethical responsibilities. While students demonstrate strong awareness of key risks such as autonomous decision-making in warfare, surveillance, labour displacement, and emotional manipulation, they show limited engagement with professional codes of ethics or structured training. Instead, ethical reflection often occurs informally, through peer discussions or media exposure. These findings highlight a consistent gap between ethical awareness and institutionalised engagement, raising questions about how future engineers will navigate the ethical challenges of AI. To address this, the paper proposes an “ethics up front” model for ethics integration that embeds reflection early in the development lifecycle, supported by participatory design, professional education, and regulatory alignment. This paper provides empirical evidence on future AI engineers’ ethical orientations and proposes a practical model for early-stage ethics integration into the practice of AI and robotics engineering.

## Introduction

Artificial intelligence (AI) and robotics are rapidly transforming how societies work, interact, and govern themselves. These technologies promise efficiency, innovation, and new capabilities, yet they also raise profound questions of fairness, accountability, sustainability, and human dignity. The ways in which engineers design and deploy AI systems will determine whether these technologies advance public good or entrench harmful consequences such as bias, surveillance, or labour displacement.

Engineering has often been portrayed as a value-neutral and purely technical practice. This view is no longer tenable. Every design decision in AI and robotics is deeply intertwined with societal, environmental, and human factors, influencing not only technical performance but also broader outcomes in privacy, security, justice, and trust [1, 2]. Ignoring these dimensions risks embedding harms into the very fabric of technology. For this reason, scholars have called for ethical awareness to be embedded from the earliest stages of technological development. Recent global initiatives to regulate artificial intelligence, including emerging national and supranational governance frameworks, reflect growing recognition of the ethical risks associated with AI and robotics. However, such regulatory efforts primarily operate at the level of compliance and oversight, underscoring the importance of understanding how ethical responsibility is formed and enacted by engineers themselves during education and early stages of design. The *ethics up front* perspective [3] emphasises that ethics must not be treated as an afterthought but as a guiding principle within design itself. By engaging with values during development, engineers can anticipate and mitigate potential harms, whether foreseen or unforeseen, before they escalate into larger societal issues. The *ethics up front* approach extends existing frameworks such as Responsible AI [4] and Value Sensitive Design [5] by shifting the locus of ethical reflection to the very start of technological ideation and professional formation. Whereas Responsible AI often operates at the level of policy, compliance, and governance (typically after systems are developed) *ethics up front* emphasises anticipatory reflection during conceptualisation, before technical pathways become fixed. Similarly, while Value Sensitive Design embeds stakeholder values within the design process, *ethics up front* complements this by focusing on the ethical capacities of the engineers themselves: fostering moral awareness, reflexivity, and participatory engagement from the moment ideas are conceived. In this way, *ethics up front* reframes ethics not as an external constraint or procedural add-on, but as an intrinsic dimension of technical creativity, professional identity, and organisational culture.

Despite the strong normative case for embedding ethics, much less is known about how future engineers, i.e. the very professionals who will design and govern AI systems, actually perceive their ethical responsibilities. Previous research suggests that while engineering education increasingly includes courses on ethics, traditional approaches such as lectures or professional codes often have limited impact, and students may engage more actively through informal discussions or media exposure than through structured training [6, 7]. This raises important questions: Do tomorrow’s AI and robotics engineers recognise the ethical dimensions of their work? How do they balance awareness of risks with institutional engagement through codes, curricula, or regulation? And what models might bridge the gap between awareness and practice?

This paper addresses these questions through a survey of 98 MSc Robotics and Applied AI students at Cranfield University. The study makes three contributions. First, it provides rare empirical evidence on how future engineers reason about the ethical implications of AI and robotics, a perspective often theorised but rarely documented in data. Second, it identifies a consistent gap between ethical awareness and institutional engagement: students recognise risks such as autonomous weapons, surveillance, and emotional manipulation, yet report limited familiarity with professional codes of ethics and formal training. Third, while recent scholarship has advanced approaches such as Responsible AI [4], and Value Sensitive Design [5], these frameworks often operate at the level of institutional policy or late-stage technological evaluation. The present study introduces the concept of *ethics up front*, which extends these traditions by emphasising ethical engagement at the earliest stages of technical ideation and education. Rather than embedding ethics retroactively into mature systems, it calls for the cultivation of ethical reflexivity within the formation of engineers themselves, before design decisions solidify. This perspective complements recent work that highlights anticipatory and participatory approaches to responsible innovation [8–11], while offering empirical evidence on how such orientations can be developed in future practitioners.

By combining empirical evidence with a normative framework, this study advances debates in AI ethics, responsible innovation, and governance. It highlights the need to institutionalise ethics as a core element of engineering practice, ensuring that emerging technologies evolve in ways that are socially responsible, sustainable, and aligned with human values. The paper begins by reviewing relevant ethical frameworks and literature in Sect. 2, followed by a detailed description of the survey methodology in Sect. 3. Section 4 presents and analyses the results, highlighting key themes related to students’ ethical awareness and attitudes. Section 5 concludes the paper by offering recommendations for integrating ethics into engineering education and professional practice. Finally, Sect. 6 outlines the study’s limitations.

## Background

Historically, engineering has been portrayed as a value-neutral and purely technical discipline. However, this perception has been increasingly challenged by scholars who demonstrate that technology is inherently intertwined with social, political, and ethical values [12–14]. Winner [1] famously argued that technological artifacts can embody politics, urging critical attention to the societal roles and consequences of technological design. In his influential essay, Winner contends that machines, infrastructures, and systems are not merely neutral tools but can actively shape social relations, institutional dynamics, and power structures. His central thesis holds that technology can both reflect and reinforce political arrangements [1].

Winner identifies two primary ways in which artifacts possess political significance. First, some technologies are deliberately designed or arranged to settle social disputes or influence behaviour, effectively functioning as instruments of governance. Such decisions may embed political biases into the built environment, often in ways that become naturalised and invisible over time. A well-known example is Robert Moses’ low-hanging bridges, which were reportedly constructed to restrict access to public spaces for lower-income communities [1, 15]. Second, certain technologies are inherently political in that their operation requires or strongly favours specific social and political configurations. For instance, nuclear weapons require centralised, hierarchical authority structures due to their inherent danger and need for strict control. Their very existence imposes an authoritarian framework, making them incompatible with democratic oversight [1]. In short, Winner advocates for conscious, participatory deliberation during technological development, emphasising that decisions about technology are also decisions about the kind of society we wish to live in.

Building on this perspective, Van de Poel [2, 16] argues that engineers, as the designers of technical systems and infrastructures, bear significant moral responsibility for the societal consequences of their work. Rejecting the notion of technological determinism, he argues that the choices made during the design process, such as determining access, control, and user behaviour, can either entrench inequality or foster justice. Technology, in this view, is never truly neutral. Its development and deployment are deeply political acts, carrying profound implications for democracy, freedom, and social justice. Recognising the politics of artifacts invites a more reflective and accountable approach to engineering and technological innovation.

Despite increased emphasis on ethics within engineering curricula, research shows that traditional methods, such as lectures on professional codes of conduct or isolated case studies, are often insufficient to develop meaningful ethical competence. Herkert [17] critiques these approaches for their limited transformative impact. Abaté [18] who makes a distinction between three categories of ethics (i.e. common morality, personal morality, and professional ethics) suggests that teaching ethics should focus on fostering students’ ethical reasoning skills rather than prescribing behaviour. Colby and Sullivan [19] emphasise that ethics education must be engaging and integrated into the technical learning environment to shape students’ moral development effectively.

Veach [20] points out that professional engineering codes of ethics are valuable but potentially insufficient or even conflicting in complex real-world situations. While useful as guidance, codes should not replace moral reasoning grounded in universal human values. Veach challenges the idea of separate ethics for different life domains. He suggests that if engineers consistently applied universal ethical principles, such as the Golden Rule, which states “Do unto others as you would have them do unto you”, there would be little need for a specialised study of engineering ethics. The term “Golden Rule”, which could be referred to as an ‘ethics of reciprocity’, began to be used widely in the early 17th century in Britain, but was known as a principle in various forms across ancient civilizations, religions, and cultural traditions [21]. Adopting ideas from moral psychology and leadership theory, Veach distinguishes moral intelligence e.g. knowing what is right, and moral competence e.g. the ability to do what is right under pressure. He argues that developing moral competence requires practice, reflection, and commitment, not just knowledge of professional rules.

There are common reasons for unethical decisions by engineers such as organisational pressure, convenience, lack of clear responsibility, normalisation of deviance, and rationalisation [20, 22, 23]. These are the factors that challenge the ethical integrity of individuals and institutions alike. So instead of teaching ethics to students Veach argues it is better to develop their moral reasoning and autonomy. He advocates integrating universal principles like the Golden Rule into all aspects of education and practice, rather than siloing them into narrow professional contexts.

Ethical behaviour is not solely shaped by formal education but is also deeply influenced by professional identity, peer culture, and informal socialisation. Stappenbelt [24] shows that students often perceive their own ethical standards as higher than those of their peers, revealing a gap between individual ideals and collective norms. Professional identity, in particular, plays a crucial role in bridging individual motivations and collective team culture, as it shapes how engineers internalise shared values and translate them into daily practices. Cech [6] argues that engineering culture often fosters disengagement from social responsibility and suggests the need to better align ethics education with the lived experiences and values of engineering students. According to Abaté, students need to be helped to systematically analyse ethical dilemmas and make morally justified decisions: “the ultimate aim of efforts to teach engineering ethics is not to produce moral engineers, but rather to instill careful clarity of insight and cogent decision-making skills” [18: p588].

Bairaktarova and Woodcock [7] examine the psychological and motivational influences on ethical awareness and decision-making among engineering students. Their study demonstrates that ethical awareness is not solely the result of formal instruction or factual knowledge but is deeply intertwined with students’ intrinsic motivation and cognitive framing. Consequently, they argue that effective ethics education should focus not only on teaching ethical reasoning skills, but also on cultivating a genuine internal commitment to ethical practice. While fostering individual ethical awareness is crucial, bridging the gap between personal motivation and collective action requires attention to the social dynamics within engineering teams.

Building on this understanding, scholars have increasingly recognised the need to address the persistent gap between ethical awareness and ethical action in engineering. One promising approach is the integration of ethical reflection into the early stages of technological design and development, where ethical implications are often first embedded. Lange et al. [10] critique traditional models of ethics training, such as compliance protocols and ethics review boards, for being too focused on individuals and implemented too late in the development process to influence core design choices or team culture meaningfully. This misalignment points to what Lange and his Google colleagues call a “principles to practices gap”: while engineers may understand abstract ethical principles, they often lack the tools to apply them effectively in real-world decision-making. Importantly, ethical failures in engineering rarely result from intentional wrongdoing. More commonly, they emerge from unexamined team norms, organisational inertia, and a narrow focus on efficiency or technical performance at the expense of broader social reflection. To address these challenges, Lange et al. propose an educational approach that goes beyond individual compliance and instead seeks to reshape collective norms within engineering teams. By embedding ethics into everyday practices through participatory methods and cultivating what they call “moral imagination”, this approach aims to make responsibility a core component of engineering culture. In doing so, it aligns ethical reflection with engineers’ intrinsic motivations and practical workflows, creating conditions for more sustainable and meaningful engagement with ethics in practice.

Taebi [3] introduces the concept of an *ethics up front* approach, which emphasises that ethical considerations must be treated not as peripheral add-ons but as integral, continuous components of the innovation process. Engineers routinely engage in tasks such as risk assessment, cost-benefit analysis, and system design, all of which involve value-laden choices. Whether explicitly acknowledged or not, decisions made throughout these stages inherently carry ethical implications. An *ethics up front* perspective thus calls for anticipatory ethical thinking: engaging with normative dimensions from the outset, rather than responding to problems only after they emerge. This proactive orientation is reflected in the Value Sensitive Design (VSD) framework, developed by Friedman et al. [25] or the Design for Value approach [3], which seek to systematically embed moral and societal values into the engineering design process. VSD operationalises the principle that technologies are not ethically neutral, but rather are shaped by, and in turn shape, the values of the societies in which they are developed. Taebi further illustrates how engineering decisions can serve ethically significant ends, using examples such as the prioritisation of values like security, sustainability, safety, affordability, and privacy. Drawing on historical cases such as the forementioned Robert Moses’ low-hanging bridges, he argues that design choices often have profound ethical and social consequences, whether intended or not.

Importantly, Taebi also highlights the ethical tensions that can arise between competing values. For example, should enhancements in public security come at the cost of fundamental liberties such as personal privacy? To address such dilemmas, he proposes applying the concept of ‘values’ as a lens for navigating trade-offs in design. Related to this is the idea of “nudging”, a concept originating in behavioural economics and social psychology [26], whereby environments are deliberately structured to encourage certain choices or behaviours. This logic underpins the design of persuasive technologies, such as seatbelt alerts that emit sound signals to urge the user to wear them [27]. There are many different mechanisms of nudging, and Caraban et al. [28] put them in six categories: facilitate, confront, deceive, social influence, fear, and reinforce. In this sense, engineering ethics extends beyond abstract principles and enters the realm of design decisions that shape user conduct, prompting reflection on how technologies can guide human behaviour in ethically desirable directions. Floridi et al. [4] further argue for a normative framework to guide AI development toward societal good. Participatory design methods, as discussed by Bødker [29], empower stakeholders and expose ethical dimensions early in the innovation cycle.

Taken together, these perspectives highlight the pressing need to integrate ethics more systematically into engineering education, professional identity, and the design process itself. While theoretical models such as *ethics up front*, value-sensitive design, and participatory frameworks offer valuable pathways, there is still limited empirical evidence on how future engineers, those currently in training, perceive, internalise, and navigate these frameworks in practice. Understanding this is critical to designing ethics education that not only informs but transforms practice. To address this gap, the following study explores the ethical awareness, attitudes, and expectations of engineering students, particularly in relation to robotics and artificial intelligence. The findings offer insight into how aspiring engineers understand their ethical roles and responsibilities, where they see barriers to ethical action, and how they envision their engagement with ethics in real-world technological development.

## Methods

### Study design

This study employed a mixed-methods survey design to examine engineering students’ ethical awareness, attitudes, and engagement with ethical issues related to robotics and artificial intelligence. The survey combined closed-ended questions, analysed descriptively, with open-ended questions analysed using thematic analysis. This design enabled the integration of quantitative patterns with qualitative insights into how future engineers articulate ethical concerns and professional responsibilities. The qualitative component of the study employed a structured, question-led thematic analysis, in which open-ended responses were analysed within analytically predefined areas corresponding to the survey design.

### Participants and sampling

Participants were MSc students enrolled in Robotics and Applied Artificial Intelligence programmes at Cranfield University. A total of 98 students completed the survey, corresponding to a response rate of 61% (98 out of 161 students invited). Of these, 78.7% identified as male and 20.4% as female. The sample was demographically diverse. Participants’ average age was 29.34 years (SD = 9.01), reflecting the presence of both full-time students and part-time students concurrently employed in industry. The sample included students from a wide range of countries, with the largest groups originating from Great Britain and Northern Ireland (32%), India (27%), and France (13%). The remainder of participants represented a wide range of countries, including China, the United States, Kenya, Pakistan, Spain, Thailand, Turkey, Lithuania, and others. Importantly, none of the participants obtained their undergraduate degree from Cranfield University, as the institution does not offer undergraduate programmes. This reduces the likelihood of institutional familiarity or bias influencing responses and strengthens the independence of the sample. The study was reviewed and approved by the Cranfield University Research Ethics Committee (CURES/14934/2022).

### Procedure

An anonymous Qualtrics online survey link was shared with all students via the Announcements feature in Canvas, the university’s Learning Management System (LMS), along with an invitation to participate. Data collection took place over three periods: January–March 2023 (*n* = 52), January–March 2024 (*n* = 36), and December–March 2025 (*n* = 10). The survey began with information about the study and its procedures, and informed consent was obtained prior to participation. Responses were anonymous and no identifiable data was collected. On average, the survey took 8.5 min to complete in a single session. Although students received the survey link before the course began, they were also verbally invited to participate once the lectures started.

### Materials

The survey explored engineering students’ perceptions of their own ethical beliefs and their views on the ethical convictions and behaviours of their peers. In addition to closed-ended questions, it included open-ended items to elicit qualitative insights. The survey was organised into three sections:

- Part A collected demographic data, including year of birth, gender identity, programme of study, and country where previous academic qualifications were obtained.
- Part B examined students’ perspectives on techno-ethical issues through seven questions. The questions were “How important is it for you to reflect on ethical issues of new and emerging technologies?”, “Do you use the following ways to inform yourself about ethical issues related to robotics or AI?”(list of ways to engage), “How likely will you be dealing with ethical or moral implications of the technologies you will be developing in the future?”, “If you would identify an ethical problem with a technology you are developing, would you speak out?”, “Are there any technologies/technological advances in robotics or artificial intelligence you have a moral objection against?”, and “In your past academic experience, how often have you engaged in class in discussions of ethical issues?” This section also included a matrix question with ten statements concerning the role of ethics and professional responsibility in engineering and technology development, using a 7-point scale (1 = *strongly disagree*, 6 = *strongly agree*, 7 = *no opinion*).
- Part C assessed students’ familiarity with professional engineering codes of ethics through six key questions. Example items included: “Have you ever read an engineering professional code of ethics?”, “How important are professional codes of ethics for your (future) profession?”, “Do you believe you will always act in accordance with the principles of the relevant professional code of ethics?” and “Do you believe that practicing engineers can realistically be expected to abide at all times by their professional code of ethics?” Several closed-ended questions were followed by open-ended prompts that allowed participants to elaborate on and contextualise their responses.

### Data analysis

Quantitative data from closed-ended survey questions were analysed using descriptive statistics to summarise response distributions and identify overall trends in ethical awareness, levels of engagement, and professional attitudes. Percentages are reported for key items to provide an overview of prevailing views within the sample. Quantitative analysis proceeded in several stages. First, participants were grouped according to (i) cultural background, distinguishing between individualistic countries (the United Kingdom, the United States of America, France, Spain, Greece, and Lithuania) and collectivist countries (India, China, Thailand, Pakistan, Nigeria, Turkey, Ghana, and Kenya); (ii) age group (18–25 versus 26 and above), with the latter category intended to capture participants more likely to have prior industry experience; and (iii) programme of study (MSc Robotics versus MSc Applied Artificial Intelligence). Second, differences between groups were examined using the non-parametric equivalent of the independent samples t-test, namely the Mann-Whitney U test, with a two-tailed significance level set at *p* = .05. Statistically significant differences are reported in the Results section.

A thematic analysis was conducted for the open-ended questions using the six-phase approach proposed by Braun and Clarke [30–32]. This involved a systematic process of familiarisation with the data, generating initial codes, identifying and reviewing themes, and refining them into coherent categories. The five identified themes correspond to the structure of the survey, as the open-ended questions were designed to elicit responses across predefined areas of ethical awareness, engagement, and professional responsibility. Thematic analysis was used to organise and synthesise patterns within these areas rather than to generate inductive themes or claim saturation, consistent with Braun and Clarke’s view of thematic analysis as a flexible method adaptable to theory- and data-driven research designs [30–32]. The qualitative findings are presented in the Results section using participants’ quotations to preserve the richness of their perspectives.

Quantitative and qualitative data were analysed in parallel and integrated during interpretation. Quantitative results provided an overview of the prevalence of specific attitudes (e.g. ethical awareness and willingness to speak out), while qualitative responses offered insight into how participants understood, justified, and contextualised these attitudes. This integrative approach enabled a more nuanced understanding of ethical engagement among future engineers than either method could provide independently.

## Results

### Engineering is not value-neutral

Participants widely recognised that engineering, particularly in robotics and artificial intelligence, involves ethical and moral considerations rather than being a purely technical activity. A large majority of participants (83%) reported that reflecting on ethical issues related to new and emerging technologies is important to them.

Most participants indicated that they expect ethical considerations to be a significant part of their future professional practice. Specifically, 43.8% reported that it is moderately likely and 36.7% that it is extremely likely that they will encounter ethical or moral implications in the technologies they develop.

When asked whether they would speak out upon identifying an ethical problem in a technology they were developing, 60% of participants stated that they would definitely do so, while a further 31.6% reported that they would probably speak out. There were no significant differences in the participant division by either culture (collectivist vs. individualistic), study subject (MSc in Robotics vs. MSc in Artificial Intelligence) or age subgroups (less than 25 vs. 26 + years old).

### Engagement with ethics

Participants reported relatively limited engagement with formal avenues for exploring ethical issues related to robotics and artificial intelligence. Most participants indicated that they had *never* attended conferences or sessions specifically focused on ethics (67%), with a further 23.4% reporting that they did so only occasionally. Similarly, 63.5% of participants stated that they had *never* engaged in online forums discussing ethical implications, with only 10.4% reporting that they did so *often* or *very often*. A further 62.5% reported *never* having participated in professional seminars on ethics, while 9.3% indicated that they did so *often*. Finally, 41% of participants indicated that they had *never* taken an academic course focused on ethics.

In contrast, participants reported more frequent engagement with ethical issues through informal and accessible sources. Over half of participants (55.1%) reported reading news articles on ethics *often* or *very often*, while academic journal articles (23.7%) and books (22%) were consulted less frequently. Informal discussions were also common: 57.8% of participants reported frequently discussing ethical issues with fellow students or colleagues, followed by discussions with friends (52.5%) and family members (41.4%).

Engagement with ethics in classroom settings was reported as limited and varied. A minority of participants (11.1%) indicated that they had *never* engaged in classroom discussions on ethical issues. Others reported participating *rarely* (34.4%) or *sometimes* (34.4%), while 20% stated that they *often* engaged in classroom discussions on ethical topics.

**Fig. 1:**
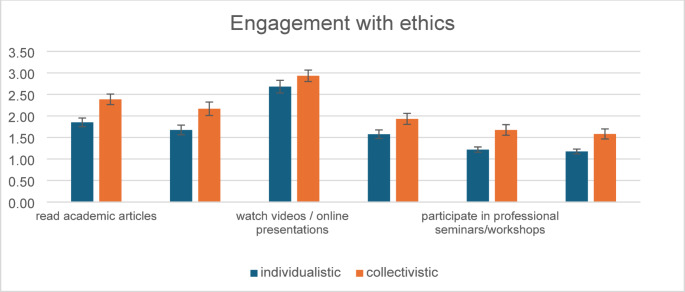
Participant engagement with ethics as a function of culture (+/− SEM)

Further non-parametric analysis using the Mann-Whitney U test revealed that engagement with ethical activities differed significantly by cultural background. Participants from collectivist cultures reported significantly higher levels of engagement than those from individualistic cultures across several activities. Specifically, participants from collectivist cultures were more likely to read academic articles on ethics (U = 669.00, *p* < .001), read books on ethics (U = 699.50, *p* = .017), discuss ethical implications in online forums (U = 648.00, *p* < .001), take academic courses on ethics (U = 787.50, *p* = .031), participate in professional seminars or workshops on ethics (U = 699.00, *p* = .004), and attend conferences or sessions specifically focused on ethics (U = 719.00, *p* = .005) (Fig. 1).

Differences were also observed by programme of study. The Mann-Whitney U test indicated a significant difference in engagement with formal ethics education between courses, with MSc Robotics students reporting participation in academic ethics courses more frequently than students enrolled in the MSc in Applied Artificial Intelligence (U = 722.00, *p* = .033; MSc Robotics Mean = 1.95, SD = 0.72, and Applied Artificial Intelligence Mean = 1.67, SD = 0.86).

**Fig. 2:**
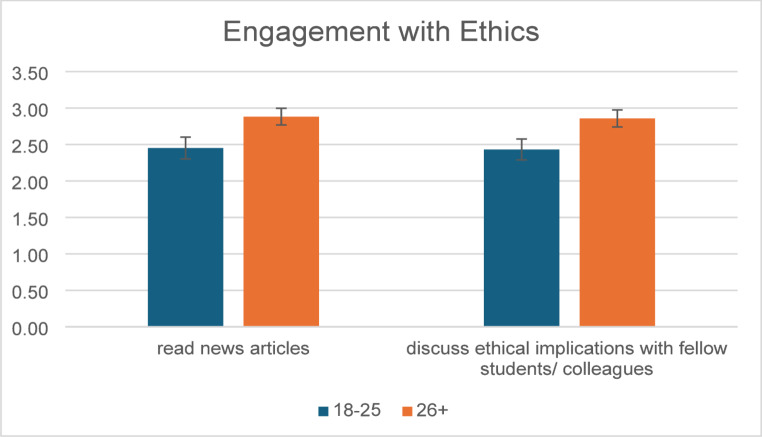
Participant engagement with ethics as a function of age group (+/− SEM)

Finally, age-related differences in engagement were identified. Participants aged 26 years and older reported significantly higher engagement with several activities compared to younger participants. Specifically, they were more likely to read news articles about ethics (U = 803.00, *p* = .030), discuss ethical implications with fellow students or colleagues (U = 809.00, *p* = .029, Fig. 2).

### Ethical awareness and moral objections

A substantial proportion of participants (82.2%) indicated that they are aware of the most pressing ethical issues associated with the technologies in which they have an interest. When asked whether they held moral objections to any technologies or technological advances in robotics or artificial intelligence, responses were distributed as follows: 39.8% responded yes, 30.6% no, and 29.9% selected “don’t know” or “no opinion”.

Participants who reported moral objections were invited to elaborate through an open-ended question. Analysis of these responses revealed several recurring areas of concern. The most prominent concerns related to artificial intelligence systems with a high degree of autonomy, particularly in high-stakes contexts such as warfare, criminal justice, and healthcare. Participants expressed strong reservations about technologies capable of making consequential decisions without human oversight. One participant articulated concern regarding the: “*decision-making capability/authority on/against/over humans in war*,* judgment*,* [and] punishment*,” while another cautioned against “*any technology that can make decisions without human intervention that can have direct long-term implications on a person’s life*,* socially or economically.*” Frequently cited examples included autonomous weapons systems, such as lethal drones, and the use of robotic autonomy in complex medical decision-making scenarios.

Another recurring concern related to technologies designed to manipulate or exploit human emotions. Participants expressed discomfort with AI systems that mimic or replace emotional or romantic relationships, particularly when such systems are used for financial gain. One participant noted: “*Those which are used for emotional manipulation*,* particularly for financial gain*,* e.g. ‘AI Girlfriend.*’”.

Concerns about surveillance and the use of personal data were also prominent. Participants raised ethical objections to technologies enabling invasive monitoring, biometric identification, and opaque data practices. Responses referenced facial recognition, predictive policing, and large-scale data collection. Illustrative comments included: “*AI in surveillance*,” “*Facial recognition*,” and “*AI used to supplant people’s identities*.” Other referenced “*predictive policing*,* autonomous sentencing of criminals*,* [and] bulk arbitrary data collection*.” Participants also highlighted the lack of transparency and explainability in AI-driven decision-making, referring to the “*lack of explainability of results*” as a significant concern.

Further concerns focused on the potential displacement of human labour and the erosion of human creativity. Participants expressed anxiety about the increasing autonomy and capability of intelligent systems and their potential social and economic consequences. As one participant noted, there is a real debate in robotics about “*the place of humans in the labour force in the coming decades.*” Others voiced unease about technologies “*that can completely replace human ingenuity*” and the broader implications of “*replacing robots with people.*”

Finally, some participants expressed ethical concerns regarding the development of artificial intelligence that could surpass human cognitive capabilities, commonly referred to as Artificial Super Intelligence (ASI). ASI remains a hypothetical concept, with the actual development of such advanced intelligence still far in the future, but the responses reflect anxieties about the potential uncontrollability of such systems and their implications for global security, autonomy, and human relevance. Illustrative quotes include references to “*Superhuman AI*,” “*Super Intelligent AI*,” and “*AI turning into sentient beings*.”

In addition to specific moral objections, participants expressed strong agreement with statements concerning engineers’ ethical responsibilities. Most participants agreed that engineers have obligations toward environmental stewardship (88.8%), should play an active role in shaping contemporary ethical debates (89.9%), and have a duty to inform the public about the ethical and societal implications of the technologies they develop (84.3%). A majority (70.8%) also agreed that ethical discussions should take place during the technology development stage rather than after problems have emerged.

The quantitative analysis revealed that participants from collectivist culture agreed more that “The general public should discuss ethics at the stage of technology development rather than complaining afterward about the problems it causes” (U = 595.00, *p* = .042, respectively). There were no significant differences in the subdivision by study subject or age.

### Adhering to professional ethical codes

While many participants acknowledged the importance of ethical considerations in their future professional practice, relatively few reported substantive engagement with formal professional codes of ethics. Only 34% of participants reported having read a professional engineering code of ethics, with older participants being significantly more likely to have done so (U = 648.00, *p* = .013; 18–25 year old group Mean = 1.49, SD = 0.73, 26 + group Mean = 1.86, SD = 0.74). Despite this limited direct engagement, a majority of participants (69.7%) indicated that membership in professional institutions such as the Engineering Council or the Royal Academy of Engineering was important. This perception was significantly stronger among participants aged 26 years and older (U = 391.50, *p* = .019; 18–25 year old group Mean = 4.39, SD = 1.42, 26 + group Mean = 5.17, SD = 0.86). In addition, 79.3% of participants stated that they believed they would personally adhere to professional ethical codes in their future practice.

Participants offered a wide range of explanations for their views on ethical adherence. Some framed compliance as intrinsic to professionalism and professional identity. For example, one participant stated: “*I will always act in line with the relevant professional code of ethics because it guides my ability to conduct myself with the utmost professionalism*.” Another participant reinforced this stance, asserting that adherence to ethical principles is *“part of my job. And as a professionally registered engineer*,* it’s part of what is expected”.* Additionally, some participants emphasised the collective agreement behind ethical codes, as reflected in the statement, *“The code of ethics was something that many people worked on and agreed that this is best for the future and humanity”.*

Ethical adherence was framed as a legal or professional necessity, often linking it to potential consequences for non-compliance. One participant stated bluntly, *“I’ll get sued otherwise”.* Similarly, another noted, *“Failure to comply with ethical standards can in some way be a breach of the law”.* Others described their adherence as shaped by institutional enforcement rather than personal conviction, as evidenced by the remark, *“In my previous job*,* I used to follow certain codes of ethics because when you don’t*,* it will backfire on you”.*

While many participants supported ethical compliance, several acknowledged that ethical codes might not always be absolute. Ethical dilemmas were a recurring theme, with participants highlighting situations where adherence to ethical principles might be challenged. One participant remarked to the question whether they were compliant, *“Most of the time yes*,* but there may be times where you will not be in accordance with the principles to achieve something in your way”.* Another noted the complexity of real-world ethical decision-making, stating, *“Certain situations require us to go out of the guidelines to solve issues that are not yes or no questions”.* Additionally, one participant expressed the unpredictability of ethical choices: *“I would hope I acted in a moral way*,* however*,* you never know until you are placed in the situations”.*

Some responses suggested that ethical considerations are often deprioritised in favour of more immediate professional or organisational concerns, in fact participants from individualist culture group had a significantly stronger agreement with statement that “The importance of profit means that companies neglect ethics” (U = 470.50, *p* < .001; Individualist group Mean = 4.21, SD = 1.63, Collectivist group Mean = 2.85, SD = 1.68). One participant candidly stated, *“We always tend to do the bare minimum. So*,* unless it is required by law*,* and when there are other pressing issues in the company*,* I doubt I will be focusing on these”.* Another highlighted the difficulty of ethical adherence in hierarchical structures, commenting, *“Following the principle in a hierarchical organisation will be quite challenging and most of the times*,* it is not under individual capability”.*

Participants expressed concern that strict adherence to ethical guidelines might hinder technological progress. One participant argued, *“In a world dominated by cutting-edge technology*,* it’s not always wise to be held back by a code of ethics as it limits the advancement of science”.* Similarly, another stated, *“Some ethics have to be followed*,* but also some ethics can be overcome by the benefit of technology”.* Finally, some participants expressed uncertainty about their ability to adhere to ethical codes, often citing inexperience or lack of familiarity. One of the younger individuals admitted, *“Not sure. Haven’t touched this field before”.* Another noted the practical challenges of ethical adherence, stating, *“If they are created*,* it is to follow them. But it might be hard to follow all these rules since we do not know them by heart”.*

Beyond professional obligations, some participants viewed ethical adherence as part of a larger moral and social commitment. One participant linked ethical behaviour to their original motivations for entering the field, stating, *“Because my original intention in studying this field was to do something good for society”.* Another emphasised the broader impact of ethical conduct, arguing, *“Ethics are there for a reason; people and the world around us are important*,* and ignoring that is selfish”.* Additionally, one participant highlighted the role of engineers in shaping societal outcomes, stating, *“As an engineer*,* you have the ability to shape the future. So*,* you should steer the future into the morally acceptable path”.*

### The self-other perception gap

A notable disparity emerged between participants’ views of their own ethical conduct and their expectations of other engineers. While 79.3% of participants reported that they believed they would personally adhere to professional ethical codes (with participants studying MSc in Applied Artificial Intelligence agreeing with the statement more than MSc Robotics (U = 574.00, *p* = .023, MSc Applier Artificial Intelligence group Mean = 4.16, SD = 0.99, MSc Robotics Mean = 3.79, SD = 0.83), only 48.3% indicated that practicing engineers in general could realistically be expected to do so consistently.

Participants provided a range of explanations for their views regarding other engineers’ ethical behaviour. Many participants expressed the belief that ethical adherence is an essential part of engineering practice. Several emphasised that ethics are embedded within the profession, stating that “*engineering operates according to standards that will most likely include ethical considerations*” and that “*the code of ethics is usually based on safety and has good reasons behind it. It should be followed*”. Others highlighted the responsibility that engineers bear, stating that “*practicing engineers should set goals to minimise ethical breaches and ensure that engineers are equipped to handle ethical challenges responsibly*”. Some participants framed ethical adherence as a fundamental expectation: “*I believe practicing engineers can realistically be expected to abide at all times by their professional code of ethics because this is the only approach that guarantees that they can avoid the several possible ethical landmines that could create problems for them and their organisation down the line*”.

In contrast, many participants highlighted challenges that may prevent engineers from adhering consistently to ethical standards. External pressures, particularly those related to commercial interests, were frequently cited. One participant remarked: “*I think in some cases not all parts of ethics are considered*,* perhaps on purpose by those looking for highest profits*”. Another noted that “*sometimes pressure gets to engineers*,* which can bend their morals*”. The tension between ethical principles and corporate goals was also highlighted: “*Ethics*,* much like principles*,* are often only worth something when they cost little to uphold. A business will not bankrupt itself over an ethical dilemma when either outcome is legally permissible. In fact*,* if it is a publicly traded company*,* the board has a legal obligation to maximise profit to shareholders*”.

Several participants emphasised personal and situational constraints affecting ethical behaviour, including self-interest and survival pressures. “*People can be selfish*,* or in compromised positions that would allow them to make decisions that may breach ethics codes*,” noted one participant. Another stated that “*people tend to focus on existence rather than ethics in most settings*”. These perspectives suggest that, while engineers may personally value ethical conduct, practical realities can impede adherence by other engineers.

The role of organisational culture and external regulatory structures was frequently cited as shaping ethical behaviour. One participant remarked, “*It really depends on the environment and the regulatory structures set up in different environments to supervise compliance*”. Another stressed that “*engineering needs to be professionalised and understood as a profession with ethics as strong as medicine or law*”. Others recognised the hierarchical constraints within organisations, as illustrated by the statement, “*Engineers are mostly constrained by the leaders who design and manage the products engineers need to implement*”.

Regulatory gaps and awareness issues were again mentioned. One participant highlighted that “*codes of ethics evolve over time*,* an engineer may not be aware of an update and so may inadvertently breach a new code*.” Another added, “*Unaware of entire code. Push by higher-ups to release products neglects the need to abide by ethical standards”.*

Ethical considerations are not always clear-cut and may involve complex trade-offs. “*Two rules of the ethics code might be opposite*,” observed one participant, reflecting the potential for conflicting ethical imperatives. Another emphasised that “*Statistically*,* it is far more likely that at some point an engineer will face a situation where they feel forced to go against their code of ethics*,* rather than never encountering that issue. Also*,* everything is a trade-off*,* so in specific scenarios rejecting the code might be beneficial in other ways*”.

Defence-related industries were frequently cited as examples of ethical ambiguity. One participant pointed out, “*Defence companies*,* for example*,* are pragmatically required for the safety of countries; however*,* they profit off lethality. It is a hard answer if they act ethically or not*”. Another participant expressed a similar sentiment: “*An example is in the defence sector: the creation of the technology actually helps with worldwide technological advancements in all sectors*,* BUT the products developed will initially be used in warfare*,* which some people may argue is for protecting and defending the country*,* but simultaneously innocent people will end up injured or dead as a result. However*,* if they don’t work on it*,* someone else will*”. There were suggestions that ethical considerations must be balanced with technological progress. One participant noted “*All things have a positive and negative impact. One cannot have one without the other*,* else there won’t be progress*”. Another stated, “*The weight of impact in the benefit of technology outweighs certain ethical impacts*”.

An overview of the five core themes identified in this Results section, together with representative participant quotations, is provided in Table 1.

**Table 1:** Core themes and representative quotes

Core theme	Description	Representative participant quotes
Engineering is not value-neutral	Participants widely recognised that robotics and AI development is inherently value-laden and likely to involve ethical and moral implications in future professional practice.	“I think it is extremely likely that I will be dealing with ethical or moral implications of the technologies I will be developing.”
Engagement with ethics	Ethical engagement occurred predominantly through informal channels such as discussions with peers and media consumption, while formal engagement through courses, conferences, or professional events was limited.	“I usually discuss ethical implications with fellow students or colleagues.”
Ethical awareness and moral objections	Participants expressed awareness of key ethical risks and raised moral objections to specific AI and robotics applications, particularly autonomous decision-making, surveillance, emotional manipulation, labour displacement, and superintelligence.	“Decision-making capability/authority on/against/over humans in war, judgment, [and] punishment.” “Those which are used for emotional manipulation, particularly for financial gain, e.g. ‘AI Girlfriend.’”
Adhering to professional ethical codes	Views on professional codes of ethics ranged from strong intrinsic commitment to recognition of ambiguity, enforcement-based compliance, and organisational constraints.	“I will always act in line with the relevant professional code of ethics because it guides my ability to conduct myself with the utmost professionalism.” “Certain situations require us to go out of the guidelines to solve issues that are not yes or no questions.”
The self-other perception gap	Participants expressed greater confidence in their own ethical conduct than in the ability of other engineers to consistently adhere to ethical standards, citing commercial pressure and organisational hierarchy.	“Sometimes pressure gets to engineers, which can bend their morals.” “A business will not bankrupt itself over an ethical dilemma.”

## Discussion

### Engineering is not value-neutral: ethical awareness as a baseline

The findings indicate that future engineers do not perceive robotics and AI development as value-neutral, but rather as inherently connected to ethical and societal considerations. High levels of reported ethical awareness and anticipated responsibility suggest that ethical reflection is already present at the level of professional self-understanding among students in these fields.

However, recognising that ethical issues exist does not, in itself, guarantee meaningful ethical engagement in practice. While participants reported strong intentions to reflect on ethics and to speak out when problems arise, such intentions are formed prior to entering organisational contexts where commercial pressures, hierarchical decision-making, and regulatory constraints may limit individual agency. Ethical awareness therefore appears to function as a baseline orientation rather than as a sufficient condition for ethical action.

From the perspective of an “ethics up front” approach, these findings are significant. They suggest that future engineers are receptive to ethical reflection at early stages of professional formation, creating an opportunity to embed ethics before design pathways, organisational norms, and technical commitments become fixed. Rather than treating ethics as a corrective mechanism applied after technological development, the results support approaches that integrate ethical reflection as a constitutive element of engineering practice from the outset.

### Engagement with ethics: the limits of informal reflection

The findings reveal a clear pattern in how future engineers engage with ethical issues: ethical reflection is widespread but largely informal. Students most frequently encounter ethics through news media and peer discussions rather than through structured educational or professional forums. While such informal engagement may foster awareness and moral sensitivity, it lacks the consistency, depth, and institutional anchoring required to support ethical decision-making in complex professional environments.

The relative absence of sustained engagement with formal ethics education and professional ethics events suggests that ethics remains peripheral within technical training, rather than being embedded as a core component of engineering practice. Classroom discussions appear sporadic rather than systematic, reinforcing the impression that ethical reflection is often treated as supplementary rather than integral to technical learning.

The observed cultural and age-related differences further suggest that ethical engagement is not solely an individual cognitive disposition but is shaped by social, cultural, and experiential factors. Participants from collectivist cultures engaged more frequently with both formal and informal ethical activities, including academic reading, public discussion, and professional events. This pattern suggests that ethical reflection may be more socially embedded in collectivist contexts, where moral reasoning is oriented toward collective deliberation and shared responsibility rather than individual discretion.

Similarly, older participants demonstrated higher levels of ethical engagement across several measures, suggesting that ethical reflection is shaped by professional exposure and situational relevance rather than developing automatically through formal education alone. Taken together, these findings reinforce the argument that ethics education must be structured and sustained, rather than assumed to emerge organically through awareness or experience.

From an ethics up front perspective, this pattern is particularly significant. If ethical engagement primarily occurs after technologies are conceptualised or through external media narratives, opportunities for anticipatory ethical reflection during design and development may be limited. Embedding ethics earlier and more consistently within engineering education could help transform informal concern into structured ethical capacity, enabling future engineers to integrate ethical reasoning into everyday technical decision-making rather than addressing it retrospectively.

### Moral objections and high-risk AI applications

The findings demonstrate that future engineers’ ethical awareness is not abstract or generic, but oriented toward concrete technological applications and their societal consequences. Participants’ moral objections clustered around a set of high-risk AI and robotics applications that are widely recognised as ethically contentious, including autonomous weapons, algorithmic decision-making in justice and healthcare, surveillance technologies, emotional manipulation systems, and large-scale automation.

Concerns about autonomy and human oversight were particularly salient. Participants consistently expressed discomfort with technologies that concentrate decision-making authority in automated systems, especially when such decisions affect life, liberty, or social standing. This emphasis suggests that future engineers are sensitive to questions of accountability, control, and moral responsibility, even when they do not frame these concerns using formal ethical terminology.

The prominence of surveillance, biometric identification, and opaque data practices reflects broader societal anxieties about privacy, explainability, and power asymmetries in AI governance. Similarly, concerns about emotional manipulation and artificial companionship point to unease with technologies that blur boundaries between human relationships and commercial exploitation. These concerns indicate that participants are attuned not only to technical risk, but also to the social and relational dimensions of AI deployment.

At the same time, anxieties about labour displacement, loss of human creativity, and artificial superintelligence reveal ethical concerns that extend beyond immediate design decisions to longer-term societal trajectories. While some of these technologies remain speculative, their presence in participants’ responses suggests that ethical reflection among future engineers is shaped by broader public discourse, media narratives, and anticipatory fears about technological futures.

From an ethics up front perspective, these findings are instructive. They suggest that future engineers already possess a substantive ethical vocabulary oriented toward risk, harm, and societal impact, but that this awareness remains largely reactive. Without structured opportunities to translate moral concern into design choices, governance mechanisms, and stakeholder engagement, ethical objections risk remaining at the level of apprehension rather than action. Embedding ethical reflection at the earliest stages of technological ideation may help bridge this gap by enabling engineers to anticipate, rather than merely respond to, ethical challenges associated with high-risk AI applications.

### Professional codes between commitment and constraint

The findings reveal a complex and often ambivalent relationship between future engineers and professional codes of ethics. Although most participants expressed confidence in their own ethical intentions and affirmed the importance of professional ethics, direct engagement with formal ethical codes remained limited. Differences between programmes indicate that students enrolled in the MSc Applied Artificial Intelligence programme were more likely to have read a professional code of ethics. While no significant differences were observed between age groups, this pattern may nevertheless be shaped by age and prior professional experience, as Applied Artificial Intelligence students tended to be older and more likely to enter the course directly from industry. Overall, these findings suggest that professional codes of ethics often function more as symbolic reference points than as practical tools guiding everyday decision-making.

Participants’ explanations reveal multiple, and sometimes competing, orientations toward ethical codes. For some, adherence was framed as an intrinsic aspect of professional identity, closely tied to notions of integrity, responsibility, and societal contribution. For others, ethical compliance was described primarily in instrumental terms, shaped by legal risk, organisational enforcement, or fear of negative consequences. These differing framings suggest that ethical adherence is not grounded in a single moral logic but emerges from a combination of personal values, professional norms, and institutional pressures.

The frequent acknowledgement of ethical ambiguity and contextual complexity further complicates the role of professional codes. Participants recognised that real-world engineering decisions often involve competing values and trade-offs that cannot be resolved through rigid rule-following. In hierarchical and commercially driven organisational settings, individual engineers may have limited capacity to act on ethical concerns, even when they are aware of relevant principles.

The differences observed between programmes further suggest that ethical engagement is shaped by professional identity formation. While students in Robotics were more likely to encounter ethics through formal coursework, students in Applied Artificial Intelligence were more likely to have read professional codes and to express confidence in adherence. This divergence implies that ethics is framed differently across technical domains, being treated as an educational topic in some contexts and as a professional compliance framework in others. Ethics initiatives may therefore need to be tailored to disciplinary cultures rather than delivered uniformly.

Concerns that ethical guidelines may constrain innovation point to a perceived tension between ethical responsibility and technological progress. While this view was not universal, its presence highlights the risk that ethics may be framed as an external constraint rather than as an enabling component of responsible design. This perception reinforces longstanding critiques in the literature that professional codes alone are insufficient to foster ethical practice in fast-moving technological fields [6, 10, 17, 20].

From an ethics up front perspective, these findings underscore the limitations of relying on professional codes as the primary mechanism for ethical governance. If ethical reflection is introduced only at the level of compliance or post hoc accountability, it risks being sidelined by organisational priorities and innovation pressures. Embedding ethical reflection earlier in education, design processes, and professional formation may help reframe ethical considerations as integral to technical decision-making rather than as obstacles to progress.

### From individual ethics to collective responsibility

The findings reveal a clear self-other perception gap in how future engineers assess ethical conduct. While participants expressed strong confidence in their own ethical intentions, they were notably more sceptical about the ability of other engineers to adhere consistently to professional ethical standards. This asymmetry aligns with prior research showing that engineering students often perceive themselves as more ethically reliable than their peers [24], reflecting broader psychological tendencies of self-enhancement and self-serving bias in ethical self-assessment [33–35].

Importantly, participants did not attribute ethical shortcomings primarily to individual moral failure. Instead, they repeatedly cited external and structural factors, including commercial pressure, organisational hierarchy, regulatory gaps, and situational trade-offs, as reasons why other engineers might struggle to act ethically in practice. This framing suggests that ethical behaviour is understood as context-dependent and institutionally constrained, rather than solely a matter of personal character or intention.

Frequent references to defence-related technologies and profit-driven decision-making further underscore the collective and systemic dimensions of ethical responsibility. Participants recognised that engineers often operate within complex socio-technical systems where ethical agency is unevenly distributed and where refusal to participate in ethically contentious work may be perceived as impractical or ineffective. Such reflections echo existing critiques of ethics frameworks that focus narrowly on individual responsibility while underplaying the role of organisational and economic structures.

From an ethics up front perspective, the self-other perception gap is particularly instructive. It highlights the limitations of approaches that place primary responsibility on individual ethical resolve or rely on post hoc mechanisms such as whistleblowing. If ethical behaviour is widely perceived as contingent on organisational culture, incentive structures, and decision-making hierarchies, then ethical governance must extend beyond individual compliance to address how teams, institutions, and design processes shape ethical outcomes. Embedding ethics earlier in technological development, education, and organisational practice offers a way to reduce reliance on individual moral resolve. By fostering shared ethical norms, anticipatory reflection, and participatory decision-making, an ethics up front approach reframes ethical responsibility as a collective endeavour, creating conditions in which ethical concerns can be raised and addressed proactively rather than only after harms have become entrenched. The core empirical findings and their implications for engineering education, professional practice, and governance are synthesised in Table 2.

The cultural differences observed in attitudes toward public ethical discussion and corporate responsibility further underscore that ethical governance cannot be reduced to individual moral resolve. Participants from collectivist cultures were more supportive of early-stage public ethical deliberation, whereas participants from individualistic cultures more strongly associated ethical neglect with profit-driven organisational structures. These differing moral framings suggest that ethical responsibility is culturally mediated and that effective governance frameworks must accommodate diverse moral logics rather than presuppose a single model of ethical agency.

**Table 2:** Summary of core empirical findings and implications

Core findings	Summary of findings	Implications/recommendations
Ethical awareness is high	Most future engineers recognise major ethical issues such as autonomous weapons, surveillance, and emotional manipulation. A large majority expect to face ethical challenges in their careers.	Build on this awareness by providing structured opportunities for ethical reflection within curricula and project work.
Engagement is largely informal	Students engage with ethics mainly through news, peers, or social media, while formal engagement through courses or professional events remains limited.	Integrate ethics throughout technical education rather than in isolated modules. Encourage applied ethics discussions in technical contexts.
Gaps in familiarity with professional codes	Only one-third of participants have read a professional code of ethics, despite most expressing confidence that they would act ethically in practice.	Strengthen ethics literacy by embedding professional codes into education, accreditation, and mentoring.
Ethical tensions and organisational pressures	Students acknowledge that commercial, hierarchical, and regulatory pressures can constrain ethical behaviour.	Promote organisational cultures that support ethical reasoning, transparent governance, and responsible innovation.
From awareness to action: the “ethics up front” model	Awareness alone does not ensure ethical practice; ethics must be embedded early in design and decision-making.	Apply an “ethics up front” framework that integrates reflection at the ideation stage, includes diverse stakeholders, and aligns technical goals with societal values.

## Conclusions

The findings of this study indicate that future engineers are generally aware of the ethical challenges associated with robotics and artificial intelligence and express willingness to take responsibility for their societal impacts. However, this awareness is not matched by consistent engagement with professional codes of ethics, structured ethics education, or institutional support mechanisms. Ethical awareness alone therefore appears insufficient for cultivating sustained ethical practice. Rather, ethics must be systematically integrated into the culture, curriculum, and everyday decision-making of engineering professions. While many future engineers appear open to incorporating ethics into their professional identity, stronger institutional structures are needed to support this engagement.

The inferential findings indicate that ethical engagement is not uniformly distributed across future engineers, but varies systematically with cultural background, age, and disciplinary context. This variation suggests that ethical capacity is socially and institutionally shaped rather than merely a function of individual moral intention, reinforcing the need for structurally embedded, culturally sensitive, and context-aware ethics education and governance frameworks.

The study identifies several areas where ethics can be embedded more effectively in engineering education and professional life. These include integrating ethics as a core component of engineering curricula rather than as an optional add-on; encouraging early-stage ethical reflection through frameworks such as *ethics up front* and participatory design; strengthening engagement with professional codes of ethics through assessment, accreditation, and mentoring; fostering workplace cultures that support open discussion of ethical concerns without fear of reprisal; and enhancing regulatory and institutional oversight so that corporate and professional incentives align more closely with societal values.

As robotics and artificial intelligence continue to shape social, economic, and political life, the extent to which these technologies empower or disadvantage communities will depend not only on technical capabilities but also on the ethical and institutional contexts guiding their development. The findings suggest that fostering ethical reflection early in engineering education and design processes can help future professionals navigate complex trade-offs and align technological innovation with societal values. Ethics should therefore be treated as an integral part of design and professional formation, rather than as a post hoc compliance exercise.

The results reinforce the value of integrating ethics from the very beginning of technology development, in line with approaches such as *ethics up front* and value-sensitive design. Grounding ethics initiatives in the lived experiences, motivations, and constraints of engineers themselves may represent a practical way to bridge the persistent gap between ethical awareness and ethical action. Engineering education should thus aim not only to cultivate moral awareness, but also to develop the competence to act ethically under pressure.

Importantly, the findings also caution against overreliance on whistleblowing or individual acts of ethical resistance as primary mechanisms for ethical governance. Many participants implicitly recognised the power asymmetries and structural constraints that shape engineering practice, particularly for early-career engineers operating within hierarchical and commercially driven organisations. Expecting MSc-level engineers to consistently challenge unethical practices through whistleblowing places a disproportionate burden on individuals with limited decision-making authority and significant professional risk. An ethics up front approach offers an alternative by reducing reliance on retrospective ethical interventions and individual moral courage. By embedding ethical reflection, value deliberation, and stakeholder engagement at the earliest stages of education and design, ethics up front helps distribute ethical responsibility across teams, organisations, and processes, rather than locating it solely in individual acts of resistance. In this way, ethical action becomes structurally supported rather than personally exceptional.

At the same time, the study highlights the structural and organisational constraints that shape ethical behaviour in practice. Engineers operate within environments characterised by commercial pressures, hierarchical decision-making, and regulatory ambiguity, which can limit individual ethical agency even among well-intentioned practitioners. Creating ethically responsible technologies is therefore not solely an individual task, but a collective institutional and professional effort. Aligning technological development with public values requires supportive organisational cultures, participatory design practices, and institutional frameworks that enable ethical reflection to translate into ethical action.

## Limitations and future directions

This study offers valuable insights into the ethical awareness and attitudes of future robotics and AI engineers, but several limitations should be acknowledged. First, the survey was conducted at a single university, which limits the generalisability of the findings across institutional, disciplinary, and cultural contexts. While the international composition of the cohort partially mitigates this limitation, broader cross-institutional and cross-cultural studies would strengthen external validity.

Second, the findings are based on self-reported data and may therefore be influenced by social desirability or self-enhancement biases, particularly in relation to ethical intentions and professional conduct. In addition, the study captures perceived attitudes rather than observed ethical behaviour in professional settings, where organisational, commercial, and regulatory pressures may shape decision-making in more complex ways.

Third, although the qualitative responses provide rich insight into participants’ ethical reasoning, they cannot fully capture the situational and contextual complexity of ethical decision-making in practice. The survey also did not include questions assessing awareness of AI-specific technical or governance standards (e.g. ISO/IEC 42001), nor did it collect detailed data on participants’ years of industry experience, limiting the ability to examine how professional exposure influences ethical engagement.

Future research could address these limitations by conducting multi-institutional and longitudinal studies to examine how ethical awareness develops over time and translates into professional behaviour. Further work could also explore engineers’ familiarity with emerging AI standards and governance frameworks, as well as empirically test the application of the “ethics up front” model within design processes, workplace cultures, and policy development.

## Data Availability

The dataset generated by the survey research during and/or analysed during the current study are available in the Zenodo repository (EU Open Research Repository), https://zenodo.org/records/17600198. 10.5281/zenodo/17600198.
